# Identification, characterization and expression analysis of lineage-specific genes within sweet orange (*Citrus sinensis*)

**DOI:** 10.1186/s12864-015-2211-z

**Published:** 2015-11-23

**Authors:** Yuantao Xu, Guizhi Wu, Baohai Hao, Lingling Chen, Xiuxin Deng, Qiang Xu

**Affiliations:** Key Laboratory of Horticultural Plant Biology (Ministry of Education), Huazhong Agricultural University, Wuhan, 430070 China; Agricultural Bioinformatics Key laboratory of Hubei Province, College of Information, Huazhong Agricultural University, Wuhan, 430070 China

**Keywords:** Citrus, Lineage-specific gene, Transcriptome, Abiotic stress

## Abstract

**Background:**

With the availability of rapidly increasing number of genome and transcriptome sequences, lineage-specific genes (LSGs) can be identified and characterized. Like other conserved functional genes, LSGs play important roles in biological evolution and functions.

**Results:**

Two set of citrus LSGs, 296 citrus-specific genes (CSGs) and 1039 orphan genes specific to sweet orange, were identified by comparative analysis between the sweet orange genome sequences and 41 genomes and 273 transcriptomes. With the two sets of genes, gene structure and gene expression pattern were investigated. On average, both the CSGs and orphan genes have fewer exons, shorter gene length and higher GC content when compared with those evolutionarily conserved genes (ECs). Expression profiling indicated that most of the LSGs expressed in various tissues of sweet orange and some of them exhibited distinct temporal and spatial expression patterns. Particularly, the orphan genes were preferentially expressed in callus, which is an important pluripotent tissue of citrus. Besides, part of the CSGs and orphan genes expressed responsive to abiotic stress, indicating their potential functions during interaction with environment.

**Conclusion:**

This study identified and characterized two sets of LSGs in citrus, dissected their sequence features and expression patterns, and provided valuable clues for future functional analysis of the LSGs in sweet orange.

**Electronic supplementary material:**

The online version of this article (doi:10.1186/s12864-015-2211-z) contains supplementary material, which is available to authorized users.

## Background

Lineage-specific genes (LSGs) are a set of genes in one taxonomic group that have no significant sequence similarity to any other lineages [1–7]. At early stage, due to the limited extent of genome sequences across many biological lineages, LSGs were simply thought to be an unexplained artifact [8]. However, with the availability of sequenced genome and transcriptome sequences from a large number of species, the number of LSGs has continued to increase and LSGs in many species have been studied, especially in microbial species [1, 8, 9]. Recently, comprehensive analysis of LSGs has been extended to plant families. Previous work has provided several hypotheses about the origin of LSGs, such as lateral gene transfer [10–12], duplication and subsequent sequence divergence [13, 14], *de novo* emergence from non-genic sequences [13–16], accelerated evolutionary rate [17] and so on. Besides, the majority of LSGs have no annotated function [2, 6]. Comparative genome analysis is an alternative way to investigate the functions of LSGs in species-specific biology and evolutionary significance such as speciation and adaptation.

Each newly sequenced genome contains a fraction of LSGs. Start with the first time the orphan genes (one species of LSGs) were discussed in yeast [15], LSGs within an increasingly number of lineages has been studied. For example, a model that orphan genes may be involved in the evolution of adaptive traits was proposed in Drosophila [3]. Analyses of LSGs in *Ascomycota* suggested that accelerated rates of gene evolution might promote the emergence of apparent orphan genes [17]. Insect-specific genes had been classified and fifty insect-specific proteins were characterized [18]. The origin of primate orphan genes had been discussed through a comparative genomics approach [6]. In plants, an early study in legume identified a number of legume-specific genes [19]. Rich data of expressed sequence tags (ESTs) and the genome sequences from a wide range of species have revealed a series of LSGs in *Arabidopsis* [2, 20, 21], *Populus* [21] and *Oryza* [21, 22].

Due to the lack of homology to any other genes, it is hard to excavate the biological functions of LSGs. Traditional approaches such as homology-based functional classifications are impossible. Nevertheless, the sequence information and genomic features of LSGs can provide some preliminary clues about the possible functions and evolution models of LSGs. With the availability of more and more genome sequences, several studies systematically identified the LSGs and their general characteristics have been observed such as shorter total gene length, fewer exon numbers, higher GC content compared with the conserved genes [2, 22, 23]. Besides, two aspects of evidences had been provided to prove that LSGs were real genes, not the incorrect artifacts of genomics. Firstly, the vast majority of LSGs have complete open reading frames and encode proteins with no putative functions according to the genome annotation [22]. Secondly, the EST and RNA-Seq data show that LSGs express temporally and spatially [23]. Comparative analysis of LSGs in the Brassicaceae family revealed that a fraction of LSGs were conserved Brassicaceae-specific genes [20]. The similar conclusion had been obtained in Poaceae family [22]. In addition, a number of LSGs present highly tissue-specific expression revealed by microarray experiment on different developmental stage of *Arabidopsis*. It is also observed that some LSGs expressed responsive to a wide range of stress conditions, which provided a hypothesis that the origin of some LSGs may be involved in adaption to the natural environment [20].

In this study, based on our recently published sweet orange genome and the preliminary identification of potential LSGs [24], we performed a comprehensive analysis on LSGs in citrus. A total of 296 citrus-specific genes (CSGs) and 1039 orphan genes were identified, and characterized by sequence features, including gene size, protein size, exon number, GC content, transcript support and chromosome location. In order to further understand the evolution and potential functions of the two sets of LSGs, we analyzed their expression pattern in callus, leaf, flower and fruit of sweet orange, and tissue-specific expressed genes had been examined by quantitative real-time RT-PCR (qRT-PCR). Furthermore, by checking the expression differences after stress treatments in callus, we also observed that part of the LSGs expressed responsive to a variety of abiotic stresses. Collectively, our results provided some valuable clues to uncover the evolutionary origins and functions of LSGs in the future.

## Results

### Identification of CSGs and Orphan genes

**Fig. 1:**
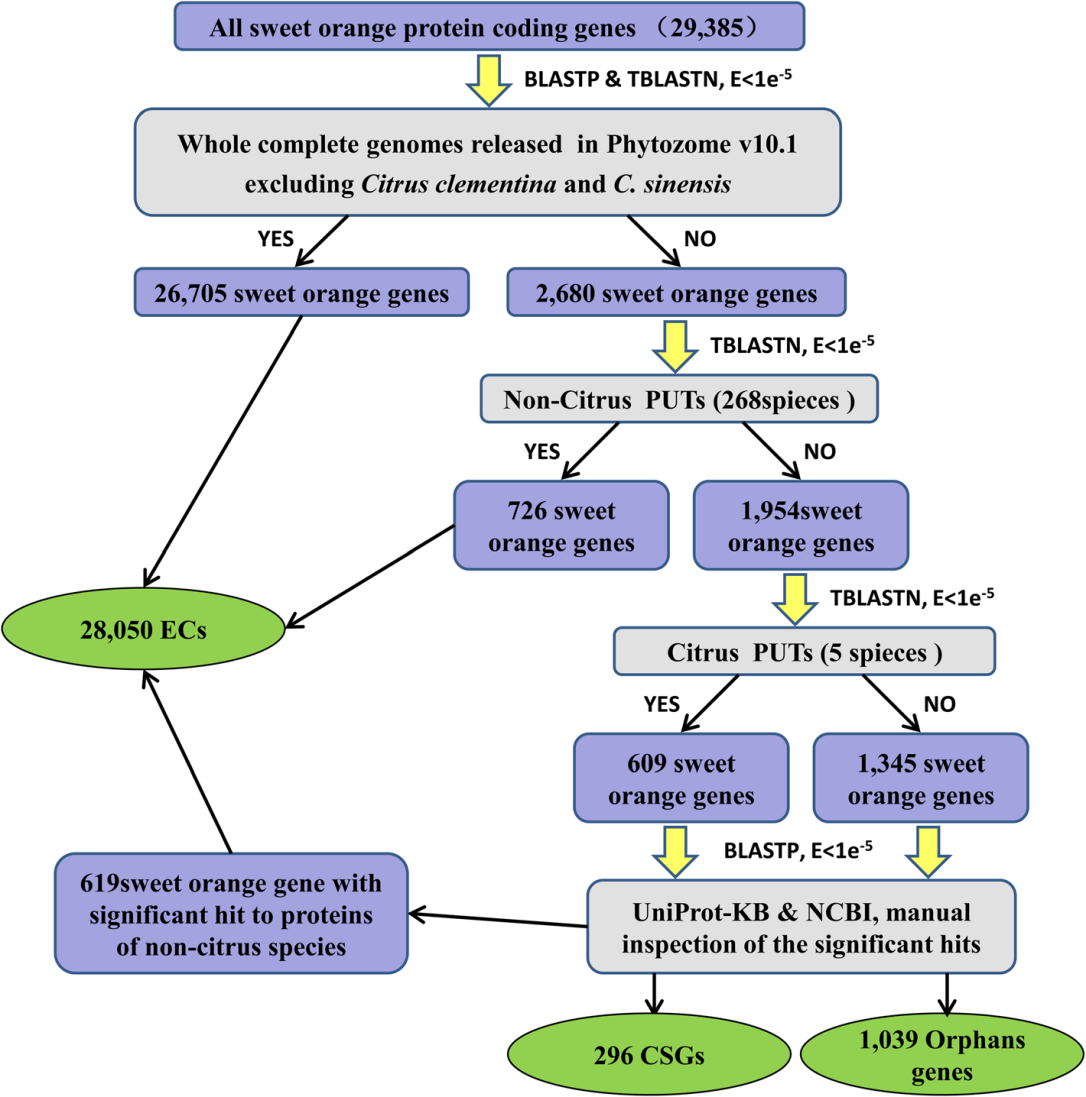
The procedure to identify citrus-specific genes (CSGs) and orphan genes in sweet orange genome

With the newly released genomes, an improved procedure was used to identify CSGs and orphan genes based on previous studies [2, 23, 25–28]. The total annotated 29,385 protein coding genes within sweet orange genome were searched against the genome sequences of 41 plant species that released in Phytozome v10.1 excluding *Citrus clementina* and *C. sinensis* by using BLASTp & tBLASTn (Additional file 1). A total of 26,705 sweet orange genes showed significant similarity (E-value < 1e-5) to at least one sequence. These sequences were defined as evolutionarily conserved genes (ECs) and were removed from further analysis (Fig. 1). Then the remained 2680 sweet orange genes that could not find any homologs in each of the genomes were used for the next step of searches, which was performed by tBLASTn analysis against the PlantGDB-assembled Unique Transcripts (PUTs) [29] from 268 non-citrus species. In this step, 726 sweet orange genes with significant similarity to at least one sequence in the non-citrus PUTs were classified into ECs. And the remained 1954 sweet orange genes with no significant similarity to either a genomic or non-citrus PUT sequence were further searched against PUT sequences from 5 citrus species or subspecies including: *Citrus aurantium*, *C. clementina*, *C. limonia*, *C. reticulata*, and *C. unshiu*. This step resulted two datasets: 1) 609 CSGs with no significant sequence similarity to sequences from the Plant Kingdom except those from citrus family, and 2) 1345 orphan genes that had no significant sequence similarity to any sequences within the Plant Kingdom (Fig. 1). In order to further eliminate false positives due to the incompleteness of the annotated protein sets and genomes, these two sets of genes were then searched against the UniProt Knowledgebase (UniProtKB) and the non-redundant protein database in NCBI using BLASTp. After manual inspection of the alignments (E-value < 1e-5), 619 genes(313 CSGs and 306 orphan genes) were assigned to the ECs. Finally, the final sets of CSGs, orphan genes, and ECs contained 296, 1039, and 28,050 sweet orange genes, respectively (Fig. 1, Additional file 2, Additional file 3).

### Characterization of CSGs and Orphan genes

Considering that functions of most LSGs were unknown and CSGs and orphan genes generally lack homology to any other genes, we characterized the genic features of CSGs and orphan genes and compared to those of the ECs (Table 1), to discern whether there are significant differences between the two sets of LSGs (CSGs and orphan genes) and the ECs. As the result, the average exon number per gene of the CSGs and orphan genes were significantly smaller than that of the ECs (one-way ANOVA; p < 0.01), consistent with the conclusion obtained in other species such as *Arabidopsis* and rice. The average exon length of the CSGs was slightly longer than that of the orphan genes and the ECs (one-way ANOVA; p < 0.01), while the average intron length of the CSGs and orphan genes were comparable to that of the ECs. Compared with the ECs, the average gene length and average protein length of the CSGs and orphan genes were signally shorter, similar to that observed in *Arabidopsis* and rice [2, 22]. However, the GC content of both the CSGs and orphan genes were significantly higher than that of the ECs (one-way ANOVA; p < 0.01), with highest for the CSGs. The higher GC content observed for the CSGs and orphan genes was in accordance with the previous report that the conserved Poaceae-specific genes have elevated GC content compared to either transposable elements or the evolutionarily conserved genes [22]. But, the observation that the CSGs had the highest GC content was contrast with the several former studies about lower GC content of LSGs in Drosophila [3], honey bee [28], and *Arabidopsis* [2, 20]. Overall, the genic feature of the CSGs and orphan genes indicated that the two sets of LSGs were distinct gene sets from the ECs.

**Table 1:** Genic features of the citrus-specific genes (CSGs), orphan genes and evolutionary conserved genes (ECs)

Feature	CSGs		Orphan genes		ECs	
	Mean (SE)	Median	Mean (SE)	Median	Mean (SE)	Median
Exons/gene	1.66 (0.92)	1.00	1.56 (0.87)	1.00	4.29 (2.88)	3.00
Exon length	381.17 (442.80)	250.50	320.14 (358.48)	221.00	322.91 (441.43)	162.00
Intron length	448.89 (452.45)	269.00	343.93 (594.69	153.00	362.67 (588.82)	171.00
Gene length	931.30 (836.43)	626.00	705.86 (781.48)	447.00	3147.41 (2844.08)	2482.00
Protein length	91.83 (51.65)	84.00	98.58 (70.61)	85.00	408.90 (315.22)	336.00
Exon GC (%)	43.53 (7.79)	42.86	43.47 (7.54)	42.57	42.28 (4.92)	42.04
Intron GC (%)	31.29 (6.88)	30.85	30.99 (7.47)	30.65	31.68 (4.92)	32.08
Gene GC (%)	42.27 (7.94)	41.28	43.00 (8.02)	42.11	38.44 (4.36)	37.73
CDS/ORF GC(%)	47.25 (7.44)	47.28	46.13 (7.29)	45.24	44.01 (3.88)	43.50
1st position GC (%)	47.42 (9.85)	47.04	45.80 (9.40)	45.52	44.65 (5.18)	43.91
2nd position GC (%)	47.99 (9.74)	47.59	46.44 (9.22)	46.21	44.82 (5.22)	44.06
3rd position GC (%)	48.62 (10.18)	47.74	47.38 (9.44)	46.73	45.01 (5.26)	44.20

**Fig. 2:**
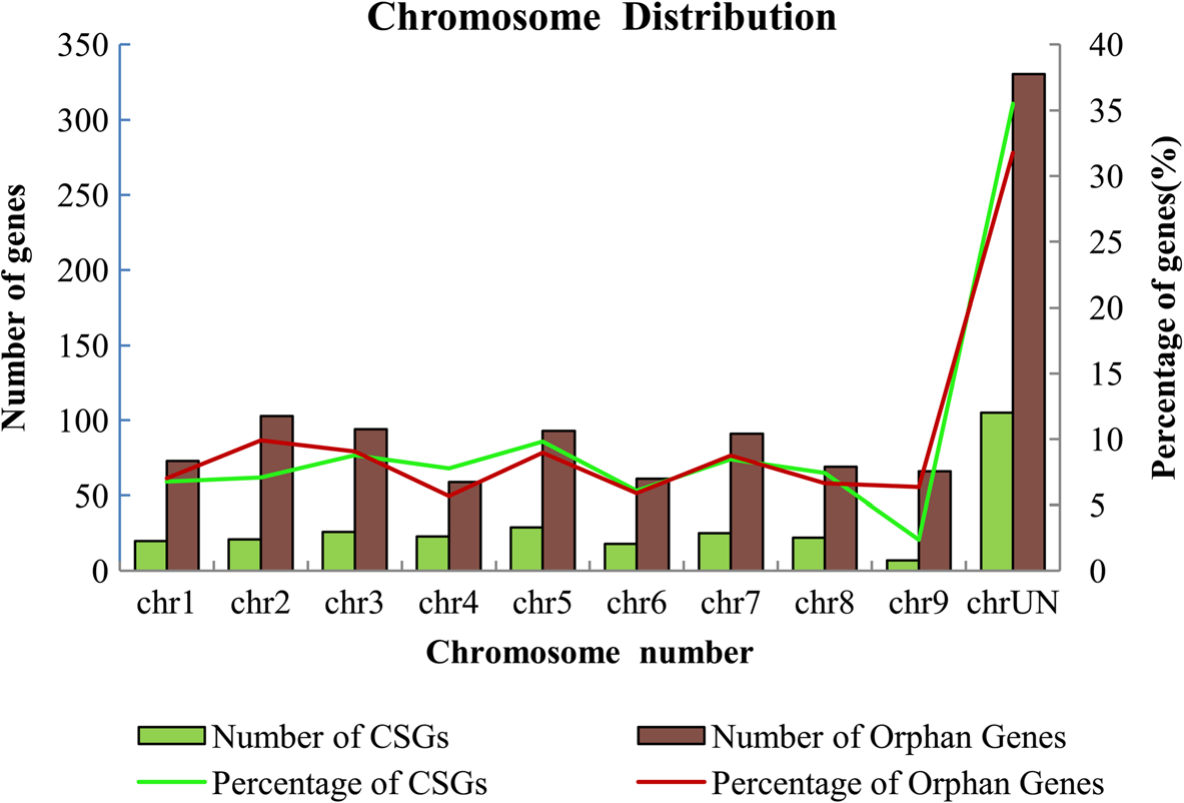
The numbers of citrus-specific genes (CSGs) and orphan genes distributed on each chromosome of sweet orange. Both numbers and percentages are shown

In order to analyze the genomic distribution of the two sets of LSGs, we mapped the CSGs and orphan genes across the 9 sweet orange chromosomes according to the information from *Citrus sinensis* genome (Additional file 4). Clearly, both the CSGs and orphan genes showed preferential distribution on certain chromosomes when compared with that of the ECs. However, the number as well the percentage of LSGs on each chromosome showed that the two sets of LSGs were distributed evenly within the different chromosomes of sweet orange (Fig. 2). Further, Spearman’s test was employed to test whether the percentage of LSGs on each of chromosome correlated with the length of the chromosomes in sweet orange. As the result of Spearman’s test, both the number of CSGs (p = 0.013, r = 0.745) and the orphan genes (p = 0.002, r = 0.842) on each chromosome correlated with the length of their respective chromosome.

### Expression pattern of CSGs and Orphan genes

As the expression pattern of a gene is often correlated with its function, RNA-Seq data derived from four different tissues of the sweet orange were used to analyze the transcript abundance of the CSGs and orphan genes. Among the two sets of LSGs, 247 CSGs and 760 orphan genes were supported by RNA-Seq data. According to the RNA-Seq data, expression profiles for different LSGs varied significantly among the four tissues. 151 CSGs and 227 orphan genes were expressed in all four tissues tested (RPKM > 0, RPKM: reads per kilobase exon model per million mapped reads), as well, 29 CSGs and 33 orphan genes showed constitutive expression (RPKM > 2 in all tissues), suggesting that the two sets of LSGs play important roles at multiple developmental stages. Interestingly, a number of LSGs showed preferential expression across the four tissues. For example, 45 CSGs and 101 orphan genes showed preferential expression in callus, 19 and 27 in leaf, 19 and 30 in flower, 22 and 25 in fruit (Table 2, Additional file 5, Additional file 6). These genes may play specialized roles in the development process of the corresponding tissues and are potential genes for further functional analysis. Above all, the expression results indicated that the majority of the CSGs and orphan genes probably participated in different biological processes in sweet orange.

**Table 2:** Tissue expression pattern of citrus-specific genes (CSGs), orphan genes and evolutionary conserved genes (ECs)

	Callus	Leaf	Flower	Fruit	Total
With tissue-specific expression					
Number of CSGs (%)	45 (42.86)	19 (18.09)	19 (18.10)	22 (20.95)	105 (100)
Number of orphan genes (%)	101 (55.19)	27 (14.75)	30 (16.39)	25 (13.66)	183 (100)
Number of ECs (%)	5249 (39.25)	2441 (18.25)	3069 (22.95)	2614 (19.55)	11373 (100)
With high expression abundance (RPKM > 2)					
Number of CSGs (%)	70 (27.88)	55 (21.91)	64 (25.50)	62 (24.70)	251 (100)
Number of orphan genes (%)	132 (34.65)	83 (21.78)	99 (25.98)	67 (17.59)	381 (100)
Number of ECs (%)	12743 (25.43)	12553 (25.05)	13426 (26.79)	11390 (22.73)	50112 (100)

Orphan genes preferentially expressed in callus when comparing with other tissues (Table 2). As shown, more than half of the tissue-specific expressed orphan genes preferentially expressed in callus. As well, the transcript abundance of all the orphan genes also conformed to this result. For the two sets of LSGs, the numbers of genes RPKM > 2 in each tissue were calculated. Clearly, the proportion of orphan genes with high transcript abundance in callus was dramatically higher than the others.

**Fig. 3:**
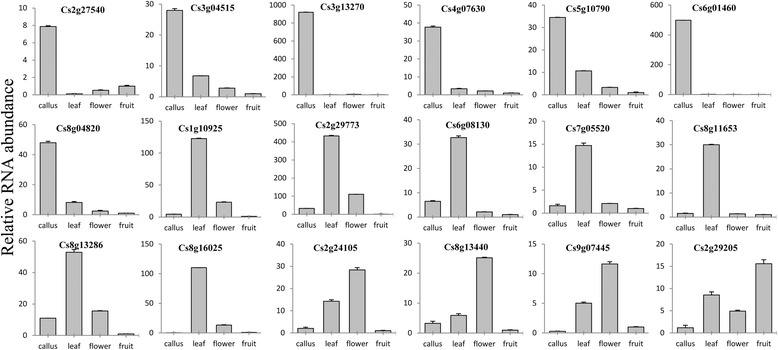
Expression patterns of 18 selected LSGs in callus, leaf, flower and fruit of sweet orange. Data were normalized to citrus β-Actin expression level. Vertical bars indicate standard deviation

In order to verify the RNA-Seq data, qRT-PCR was performed on four different tissues for 18 selected LSGs (4 CSGs and 14 orphan genes). As shown in Fig. 3, the genes showed very distinct tissue-specific expression patterns, which were in accordance with the RNA-Seq data perfectly. In detail, a subset of seven genes (*Cs2g27540*, *Cs3g04515*, *Cs3g13270*, *Cs4g07630*, *Cs5g10790 Cs6g01460* and *Cs8g04820*) among the 18 LSGs showed extremely high expression in callus, and another seven genes (*Cs1g10925*, *Cs2g29773*, *Cs6g08130*, *Cs7g05520*, *Cs8g11653*, *Cs8g13286* and *Cs8g16025*) specifically expressed in leaf, which were exactly the same as the RNA-Seq data. As well, consistent with the RNA-Seq data, three genes (*Cs2g24105*, *Cs8g13440* and *Cs9g07445*) highly expressed in flower and the gene *Cs2g29205* preferentially expressed in fruit.

### Identification of CSGs and Orphan genes expressed responsive to abiotic stress

**Fig. 4:**
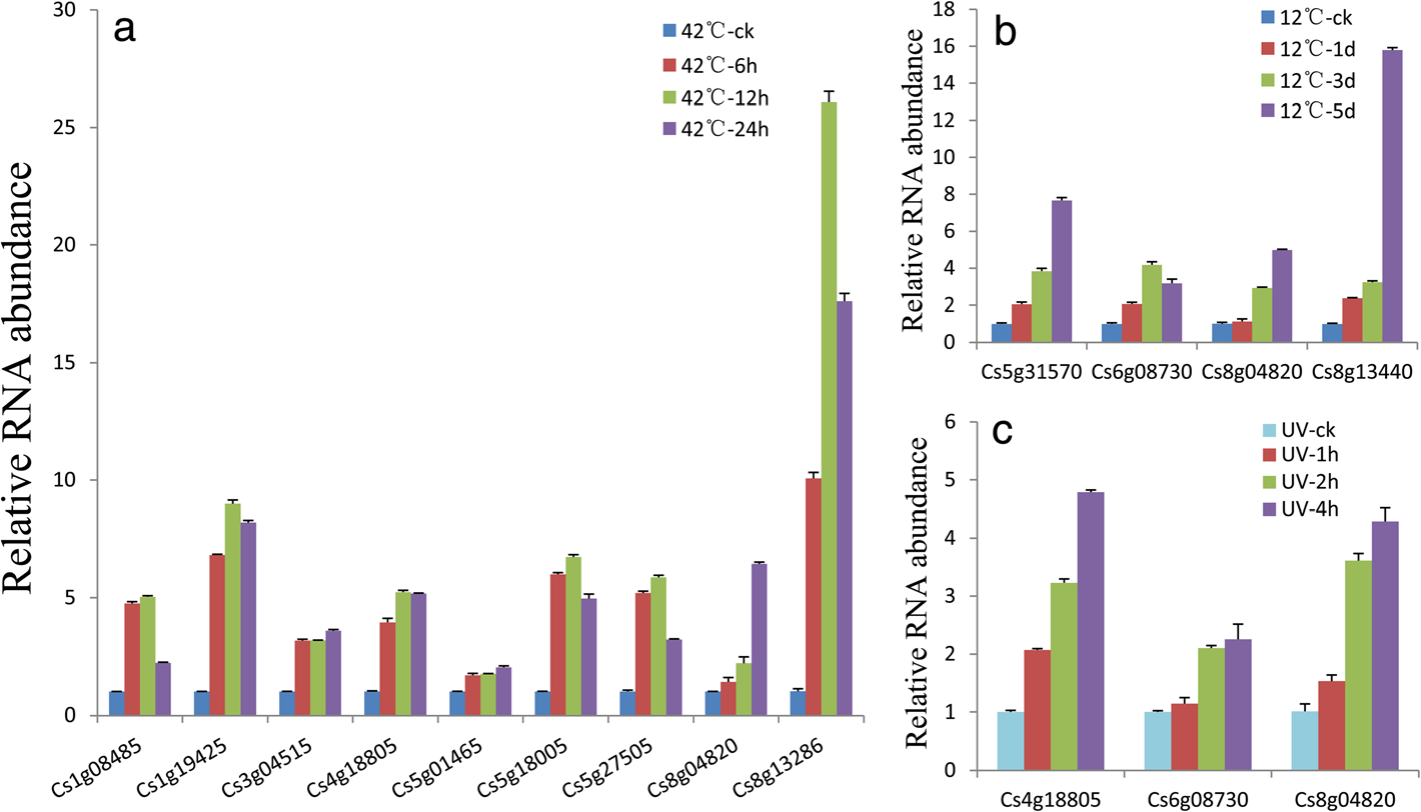
Expression patterns of LSGs responsive to abiotic stress. **a** Expression patterns of 9 LSGs under heat (42 °C) condition for 0 (ck), 6, 12, 24 h. **b** Expression patterns of 4 LSGs under cold (12 °C) condition for 0 (ck), 1, 3, 5 d. **c** Expression patterns of 3 LSGs under ultraviolet light for 0 (ck), 1, 2, 4 h. Data were normalized to citrus β-Actin expression level. Vertical bars indicate standard deviation

In order to investigate the potential roles of LSGs involved in environment adaptation, qRT-PCR was performed to determine the expression patterns of the two sets of LSGs in sweet orange callus by using different abiotic stress treatments. The callus of sweet orange was treated under three different stress conditions: cold (12 °C), heat (42 °C) and ultraviolet light (UV). A total of 47 LSGs with high expression in callus (RPKM > 2) and another randomly selected 35 LSGs were checked. Among all these genes, 12 genes (3 CSGs and 9 orphan genes) were expressed responsive to abiotic treatments (Fig. 4). Among the 9 LSGs which were up-regulated under heat stress (42 °C) condition, the gene *Cs8g13286* expressed dramatically higher than the others, which may indicate that it is likely associated with high temperature stress response (Fig. 4a). Under cold stress condition (12 °C), the majority of the LSGs were generally down-regulated. Nevertheless, there were still four LSGs up-regulated in response to low temperature and expressed at high level (Fig. 4b). Potentially, these genes were involved in cold stress tolerance. Three LSGs were significantly induced by UV (Fig. 4c). Surprisingly, the gene *Cs8g04820* was simultaneously up-regulated by the three stress treatments. Both the cold and ultraviolet light stress promoted the expression of the gene *Cs6g08730*. And the gene *Cs4g18005* was induced by both the heat and ultraviolet light stress. This result further suggested that the three genes played vital roles in stress tolerance. The above results clearly indicated that part of the CSGs and orphan genes expressed responsive to abiotic stresses.

## Discussion

Our previous study identified a preliminary set of 1691 CSGs based on the genome of sweet orange and its comparative analysis with 22 other plant species available at December 2011 [24], but as a result of rapidly increasing number of genomes have been published, it needs to be updated and characterize the sequence characteristics, gene expression patterns and potential functions. In this study, 41 genome sequences and 273 PUTs data were used to detect citrus homologous genes. In addition, the latest version of both the UniProt-KB and the current non-redundant protein database in NCBI were also used in this study. A total of 1335 LSGs in citrus were obtained in this study, similar to that reported in *Arabidopsis* [2]. An independent method using the position-specific iterated BLAST analysis suggested an overlap of 98.8 % the genes, suggesting reliability of the LSG genes identified in this study. For the identification of LSGs, the richer the genome data available, the less the false positives will be. Although there may be still some false positives in our result due to the limitation of the available genome database, our identification of the CSGs and orphan genes constitute an important step toward identification of novel genes in citrus. In the future, the LSGs in citrus could be even more accurate when the genomes of species closely related to citrus and Rutaceae are available.

Accumulating studies shows that the average length of younger genes (LSGs) is shorter than that of ECs [2, 18, 22, 23, 27, 30, 31], our data in citrus also comply with this observation. And it is reasonable to speculate that the fewer numbers of exons per gene and higher percentage of intronless LSGs lead to this phenomenon. As for the result in this study, 57.1 % of CSGs and 60.1 % of orphan genes had only one exon, while the percentage of the single exon ECs was 20.1 %. The dramatic enrichment of the intronless genes in the LSGs set compared to ECs probably resulted from recent lineage-specific expansion, which might create some new genes via retrotransposition [32, 33]. At the same time, both the CSGs and orphan genes had elevated GC content, which was consistent with previous reports that high GC content class was enriched with intronless genes in plants [34, 35], coincided with the hypothesis. Although the chromosome distribution informations of almost a third of the LSGs were unknown, more than two-thirds of them were supported by RNA-Seq data, proving that they were really genes rather than artifacts of genome annotation. Collectively, the distinct characterization of the CSGs and orphan genes with fewer exons and elevated GC content may be involved in their initial formation.

Due to the limitation of the genome sequences in citrus, origins of the LSGs are still unclear. Previous studies indicated that new genes can be created by lateral gene transfer, gene duplication, exon shuffling, retrotransposition, gene fusion/fission, mobile element, *de novo* origination and so on [4, 32, 36]. For the LSGs identified in this study, one possible mechanism is lateral gene transfer. In the last step of our pipeline (Fig. 1), the two sets of genes were searched against the UniProt Knowledgebase (UniProtKB) and the non-redundant protein database in NCBI and 619 genes were identified with sequence similarity to other species which are not available in our study. This big number suggests that lateral gene transfer may be one source of LSGs. Another important source of LSGs might be gene duplication. A number of CSGs and orphan genes were found to be located in segmentally duplicated regions of sweet orange genome, which indicted that these genes might arise from duplication and rearrangement processes followed by fast divergence. Potentially, there may be some other mechanisms for creating LSGs. A comprehensive and systematic analysis of the origins of LSGs relies on the increasing number of genomes in citrus.

Expression analysis is a feasible and effective way to detect the potential functions of the LSGs. RNA-Seq is a recently developed method for transcriptome profiling that uses deep-sequencing technologies [37]. Here, we used the RNA-Seq data from four different tissues to quantify the LSGs and highlighted several valuable properties of he CSGs and orphan genes. The small part of the two sets of LSGs presented constitutive expression may play roles as housekeeping genes, of which the gene products were necessary for the maintenance of the basal cellular function during the whole life of citrus [38]. Likewise, it is likely that those highly tissue-specific expressed genes are associated with specific phenotype or special physiological process in callus, leaf, flower and fruit. Interestingly, the orphan genes were observed to be preferentially expressed in callus. It may be explained in three aspects. Firstly, the embryonic callus is pluripotent tissue that is a stem-cell like tissue of plant which can be induced into different organs *in vitro*. The callus used in this study was subcultured for more than 30 years and is still with strong embryonic capability. The orphan genes that highly expressed in callus are probably necessary to maintain the long-time lasting pluripotency. Secondly, the orphan genes were specific to sweet orange. Coincidentally, the embryogenetic ability of sweet orange callus is stronger than the other 5 species used in this study (*Citrus aurantium*, *C. clementina*, *C. limonia*, *C. reticulata*, and *C. unshiu*) [39]. Thus, it is reasonable to speculate that some orphan genes may be related to the strong embryogenetic ability of sweet orange. Thirdly, callus can be induced by various biotic, abiotic stimuli and phytohormones [40], which suggests that part of the orphan genes may be involved in defense system. As for the rest of the genes without RNA-Seq data support, there may be two or more cases. On one hand, as the limitation of the RNA-Seq data for only four different tissues, part of those LSGs probably expressed in other specific tissues that were not quantified. On the other hand, it is possible that a small amount of genes do not express but act as regulatory roles. These hypotheses remained to be verified in the future.

Previous studies showed that some LSGs played important roles in tackling with extreme environmental conditions [20, 21, 41, 42]. Based on the microarray data, a number of LSGs responsive to different stimuli such as cold, drought, heat, oxidative, biotic stress were highlighted [20, 21]. For citrus, temperature and light are two important environmental factors that obviously affect the growth of citrus [43–45]. So we checked the gene expression of the two sets of LSGs in callus under cold, heat and UV treatments and 12 genes were observed to be stimulated by abiotic stresses. We speculate that these stress-responsive LSGs are related with the adaptation processes to maintain alive during the extreme environmental conditions. As such, knowing the functions of these LSGs is valuable to understanding the molecular mechanism of plant adaptation. Future gene function experiments including over-expression and gene silencing are necessary to validate the functions of these LSGs. Especially, three genes (*Cs4g18005*, *Cs6g08730* and *Cs8g04820*), which expressed responsive to two or more different stress conditions, have been selected for function studies through over-expression and RNAi knockdown strategy.

LSGs are thought to be responsible for the evolution of lineage-specific phenotypes and adaptive innovations [32]. Although there seems to be no exact information about the function of the two sets of LSGs, several useful clues still pointed out the future exploration aspects. Firstly, some LSGs may regulate specific phenotypes or special physiological processes, considering quite a few CSGs and orphan genes with distinct tissue-specific expression. Fortunately, a recent research showed that most of the LSGs were involved in special physiological characteristics such as energy metabolism, vitamin C metabolism, sugar-related metabolism and secondary metabolism in jujube [46]. Secondly, a set of CSGs and orphan genes were examined to be related to abiotic stress response, which was consistent with other species such as *Arabidopsis*, rice and zebrafish [2, 18, 23], calling a emerging model to explain the evolutionary development of LSGs. Further experiments are necessary to confirm whether these genes can enhance the plant tolerance to external stimuli. Thirdly, there is evidence to suggest that some genes function in transcription level as a non-coding RNA [47, 48]. Among the CSGs and orphan genes, 5 genes (*Cs1g09600*, *Cs1g09635*, *Cs1g09665*, *Cs4g19605* and *Cs8g13286*) were reported as potential target genes of microRNAs in sweet orange [49]. As thus, not all the LSGs are protein coding genes. Both the transcription and translation level are needed to be considered for researching the function of LSGs.

## Conclusion

We have characterized two sets of LSGs, CSGs and orphan genes, which are specific to citrus and sweet orange, respectively. Expression pattern analysis of the two sets of genes indicated that some LSGs played special roles in particular tissues. In addition, part of the CSGs and orphan genes expressed responsive to abiotic stress. This study provides a firm ground of citrus specific gene resources and useful clues for future dissection of the functions of LSGs to understand the specific biology in citrus.

## Methods

### Sequence data sets

The genome and proteome sequence of sweet orange was obtained from *Citrus sinensis* genome (http://citrus.hzau.edu.cn/orange/, Orange genome Annotation Project). To identify CSGs and orphan genes (sweet orange specific genes),whole the complete genomes excluding *Citrus clementina* and *C. sinensis* released in Phytozome v10.1 were used in this study (Additional file 1: Table S1). All the genomes were downloaded from Phytozome v10.1 (http://phytozome.jgi.doe.gov/pz/portal.html) on December 11, 2014. The PUTs from 273 plant species (268 Non-Citrus PUTs and 5 Citrus PUTs) were downloaded from PlantGDB (http://www.plantgdb.org/prj/ESTCluster/progress.php) on December 11, 2014. UniProtKB (Release 14.6) was downloaded from UniProt ftp://ftp.ebi.ac.uk/pub/databases/uniprot/knowledgebase/.

### Homologous sequences search

The two sets of LSGs within citrus were identified in a pipeline (Fig. 1) based on a homolog search using BLASTp and tBLASTn with an e-value cutoff of 1e-5 [2, 18, 23]. We classified the citrus genes into three categories: ECs, CSGs, and orphan genes. Here, orphan genes refer to genes for which we could not find homologs in any other species. CSGs include genes for which we could find at least one homolog in citrus, but no homologs anywhere else. ECs were genes with at least one homolog outside the group of citrus.

### Position-specific iterated BLAST analysis

In order to verify the reliability of the LSGs identified in this study, both the 296 CSGs and 1039 orphan genes were searched against the genome sequences of 41 plant species that released in Phytozome v10.1 excluding *Citrus clementina* and *C. sinensis* (sweet orange) by using the position-specific iterated BLAST [50]. The LSGs which showed significant similarity (E-value < 1e-5) to at least one sequence were considered to be false positive.

### Genic features

To observe the characteristics of the LSGs, the whole genome information of sweet orange was downloaded from *Citrus sinensis* genome (http://citrus.hzau.edu.cn/orange/). Then Perl scripts was used to calculate gene length, protein length, number of exons, and GC content of the CDS, gene, and three codon positions. We used one-way ANOVA to determine significant differences between the different sets of LSGs and the ECs. The chromosome localization information was extracted from the chromosome sequences.

### Expression analysis of CSGs and Orphan genes

The RNA-Seq raw data from four tissues (callus, leaf, flower and fruit) were derived from our genome database [24]. The expression level of CSGs and Orphan genes was calculated as reads per kilobase exon model per million mapped reads (RPKM). The criteria applied to filter out the genes with preferential expression in each tissue were: (1) the expression abundance in at least one tissue >2 RPKM; (2) the highest expression level in one tissue >2-fold-change than at least one of the other three tissues.

### Plant materials and treatments

The four different tissues of Valencia sweet orange (*C.sinensis cv. Valencia*) were separately collected. In the stress treatment experiment, the sweet orange callus growth in good condition (15 days after successive transfer culture in a growth chamber at 25 °C) were subjected to: low temperature (12 °C) treatment for 0, 1, 3, 5 d; high temperature (42 °C) treatment for 0, 6, 12, 24 h; UV (12 W/220 V,302 nm) treatment for 0, 1, 2, 4 h in MT solid medium. All samples were immediately frozen in liquid nitrogen and store at −80 °C until used.

### RNA isolation and q RT-PCR

Total RNA from different tissues was extracted using RNA extraction kit (RNAiso Plus, TaKaRa). RNA quality was monitored by gel electrophoresis and the measurement of the A260/A280 ratio. For cDNA synthesis, 1.0 μg RNA was reverse-transcribed using Maxima H Minus First Strand cDNA Synthesis Kit (Thermo Scientific) and oligo-dT primers according to the manufacturer’s procedure. Primer pairs were designed to amplify specific CSGs and Orphan genes using Primer Express 3.0 software (Applied Biosystems, Foster City, CA, USA). The primer sequences were shown in detail in Additional file 7. QRT-PCR was conducted on ABI 7900 Real Time System (Applied Biosystems) using SYBR Green PCR Master Mix (Applied Biosystems). The reactions were performed with the following cycling profile: 50 °C for 2 min and 95 °C for 1 min, followed by 40 cycles of 95 °C/ 15 s, 60 °C/ 60 s. Melting curve analysis was performed to verify the specificity of the amplicon for each primer pair. With the citrus β-Actin gene as the internal reference gene, relative gene expression values were calculated using the 2-ΔΔCt method [51].

### Availability of supporting data

All accession numbers are available in Additional file 2 and Additional file 3. The RNA sequence data supported this study have been provided in Additional file 5 and Additional file 6.

## Additional files

Additional file 1: Table S1. List of the 41 genomes released in Phytozome v10.1. The version, common name and source of all the genomes are provided. (XLS 23 kb) Additional file 2: Table S2. List of citrus-specific genes (CSGs). The gene accession, chromosome location, GC content of all the citrus-specific genes (CSGs) are provided. (XLS 56 kb) Additional file 3: Table S3. List of orphan genes. The gene accession, chromosome location, GC content of all the orphan genes are provided. (XLS 163 kb) Additional file 4: Figure S1. Chromosome distribution of citrus-specific genes (CSGs) and orphan genes in sweet orange genome. The nine sweet orange chromosomes are shown with the CSGs and orphan genes plotted in blue and red rectangle. The width of rectangle indicated the length of the genes and the height of black oblong indicated the length of the chromosome/scaffold. The chromosome numbers and gene accessions are indicated. The left “+”and right “-” of each chromosome mean the sense strand and antisense strand. The genes with no chromosome information were mapped to the putative chrUn. (PDF 1407 kb) Additional file 5: Table S4. RNA-Seq data of citrus-specific genes (CSGs) in four sweet orange tissues. (XLS 118 kb) Additional file 6: Table S5. RNA-Seq data of orphan genes in four sweet orange tissues. (XLS 191 kb) Additional file 7: Table S6. Primer sequences used in qRT-PCR. (PDF 32 kb)

## Abbreviations

LSGs: Lineage-specific genes
CSGs: Citrus-specific genes
ECs: Evolutionarily conserved genes
EST: Expressed sequence tags
qRT-PCR: Quantitative real-time RT-PCR
PUT: PlantGDB-assembled Unique Transcript
RPKM: Reads per kilobase exon model per million mapped reads
UV: Ultraviolet light
