# Supplementary figures and images for: Identification, characterization and expression analysis of lineage-specific genes within sweet orange (Citrus sinensis)
Source: BMC Genomics. 2015 Nov 23;16:995. doi: 10.1186/s12864-015-2211-z (PMC4657247; doi:10.1186/s12864-015-2211-z)

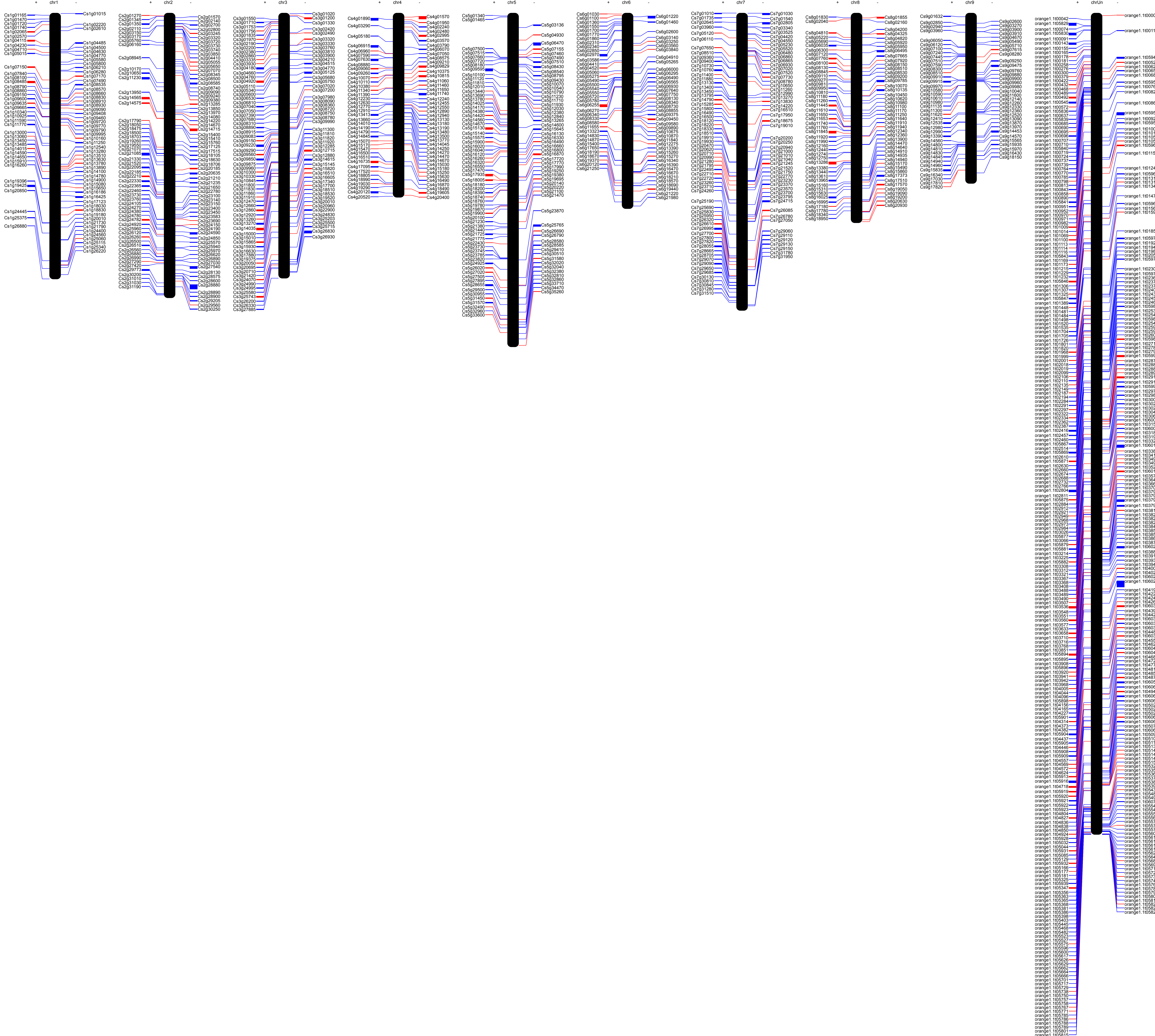

Supplement: Additional file 4: Figure S1. — Chromosome distribution of citrus-specific genes (CSGs) and orphan genes in sweet orange genome. The nine sweet orange chromosomes are shown with the CSGs and orphan genes plotted in blue and red rectangle. The width of rectangle indicated the length of the genes and the height of black oblong indicated the length of the chromosome/scaffold. The chromosome numbers and gene accessions are indicated. The left “+”and right “-” of each chromosome mean the sense strand and antisense strand. The genes with no chromosome information were mapped to the putative chrUn. (PDF 1407 kb) [file 12864_2015_2211_MOESM4_ESM.pdf]
