# Supplementary material for: Identification, characterization and expression analysis of lineage-specific genes within sweet orange (Citrus sinensis)
Source: BMC Genomics. 2015 Nov 23;16:995. doi: 10.1186/s12864-015-2211-z (PMC4657247; doi:10.1186/s12864-015-2211-z)
Supplement: Additional file 7: Table S6. — Primer sequences used in qRT-PCR. (PDF 32 kb) [file 12864_2015_2211_MOESM7_ESM.pdf]

Table S6 Primer sequences used in qRT-PCR

Primer Name	Primer Sequence	Primer Name	Primer Sequence
Actin-L	CCAAGCAGCATGAAGATCAA	Cs8g04820-F	GAGGAAGAGGATGATGATGGAA
Actin-R	ATCTGCTGGAAGGTGCTGAG	Cs8g04820-R	ACAACAGGAGGAAACAAGGACA
Cs6g08130-F	TAGCTTTTGCATGCGTGGAT	Cs6g01460-F	TTCTGTTTCTGCTAAGGCTCTGA
Cs6g08130-R	CGGTCCCATGTCAATCTCAAC	Cs6g01460-R	TCCTTCATAGCATCCTCTTCCAT
Cs7g05520-F	CCCGTCTCTCTGTTTCCTCAGT	Cs4g07630-F	TCAGCACTAAGTTGAGCCAGC
Cs7g05520-R	TCGACATAGCCCATAAAGCAAA	Cs4g07630-R	GGCGAAGAGAAAGAGAGGTCA
Cs8g11653-F	CTTCGCTTGGGAGGTTCTTTT	Cs3g13270-F	TTTTATGCTCCGCCCAAGTAA
Cs8g11653-R	TCATCAAACAGGGAAGCTGAAA	Cs3g13270-R	TGGGTCTACGCCTTCCTCAT
Cs8g13286-F	GTGTGAGCTGCCCCAAATG	Cs5g10790-F	GAAGACTCGAAAGACACAATGGAA
Cs8g13286-R	GCGTCCGCCCAATTAATCT	Cs5g10790-R	AGCCACTTTTCTCCCGTTGA
Cs2g29773-F	CTATAGTGTGCAATAGCCGCAAA	Cs1g19425-F	GCCAAAGCAAGTCCCAACA
Cs2g29773-R	GAGGAACAAAGCACCGATCAA	Cs1g19425-R	TTGTGAAGCAACCAGCACCTT
Cs3g04515-F	GCCTTATCGATGGCAACCA	Cs4g18805-F	GGAGAGCACTTTGTGTTGTTTGTT
Cs3g04515-R	GAAGATCGAAGACATGGGCAAT	Cs4g18805-R	TCTGTTTGGCTTCTGAGAAAACG
Cs2g27540-F	CCGCCGTTTGTAATTTCATCTC	Cs5g27505-F	GGTGATACCCCTTTTGGCTTAGA
Cs2g27540-R	CCCCTGAAGGCTGAAGCTATT	Cs5g27505-R	CAAGAGAAGAAGTCCCAATGCA
Cs8g13440-F	GGCCCTCGTTGTCATATTGC	Cs1g08485-F	CCTCAAAGAATCCAACCGCTAT
Cs8g13440-R	TCCACCAAAGCATTCATTTCC	Cs1g08485-R	CAAGGGCGACAACAGCACTA
Cs1g10925-F	ATCTGGTGATGAGGCCTTCAA	Cs5g18005-F	CAGCTAACGCATCATCAAAACTG
Cs1g10925-R	CCAGCGGATCCAAATTGTTATT	Cs5g18005-R	TCCAACAATGATCCACGACATT
Cs8g16025-F	CGGGTGTAGCATCATCAAGATTC	Cs6g08730-F	CAGGAGGAGAGGAAAGATTTTGAG
Cs8g16025-R	GTGCTTCGCAAGAGATTATCACA	Cs6g08730-R	TGCTCCTTCTTACTCTGGAGTTGA
Cs2g24105-F	CCCAGCAGCTACAATGATGAAG	Cs5g01465-F	CATTAGCGGTTGTGTCTGTGTG
Cs2g24105-R	CCGTGAAGAACAAGAAGACAGAAA	Cs5g01465-R	CGACTTCGTTTTGCTTTTCTTC
Cs9g07445-F	AGTATGTGCAGCTGGAGGCTTAC	Cs5g31570-F	TAGGAGGGTTCTGTGGCTTCA
Cs9g07445-R	CAGGAGAACACACCAAAAGAATGA	Cs5g31570-R	GCGTAAAGAAGTATTCCCAAAGAC
Cs2g29205-F	CCCCCAACTACAACAACGATGT		
Cs2g29205-R	CCCTGCATTGCCCGTAAA		
